# The Impact of Self-Relevance on Preschool Children’s Sharing

**DOI:** 10.3389/fpsyg.2019.01028

**Published:** 2019-05-28

**Authors:** Wenjie Zhang, Songmei Xiang, Hongmei Dai, Mengmeng Ren, Yuqi Shen, Wei Fan, Yiping Zhong

**Affiliations:** ^1^Department of Psychology, School of Education Science, Hunan Normal University, Changsha, China; ^2^Cognition and Human Behavior Key Laboratory of Hunan Province, Hunan Normal University, Changsha, China; ^3^The Second Kindergarten of Yuelu District Preschool Education, Changsha, China; ^4^Department of Pediatrics, The Third Xiangya Hospital of Central South University, Changsha, China

**Keywords:** self-relevance, recipient, resource sharing, preschool children, dictator game

## Abstract

This study was designed to investigate the impact of self-relevance between preschool children and recipients on children’s sharing behavior in dictator games using a forced-choice resource distribution paradigm. Experiment 1: A total of 75 children aged 3–6 years were evaluated in a first-party situation in which they were distributed as recipients and dictators and shared resources with distracting recipients with different extents of self-relevance under three different payoff structures, including non-costly, costly, and envy structures. Children could choose between a sharing option and a non-sharing option. The results showed that, in a first-party situation, children aged 3–6 years old typically share more resources with highly self-relevant recipients (friends) than with moderately self-relevant recipients (acquaintances) and lowly self-relevant recipients (strangers) and that they share more resources with moderately self-relevant recipients (acquaintances) than lowly self-relevant recipients (strangers). Experiment 2: A total of 62 children aged 3–6 years old were evaluated in a third-party situation in which they were distributed not as recipients but only dictators, making decisions between the options of sharing more or sharing less with distracting recipients who had different extents of self-relevance under three different payoff structures, such as non-bias, high self-bias, and low self-bias. The results showed that, in a third-party situation, children typically share in a similar manner to that of Experiment 1, meaning that children display selective generosity and that the self-relevance between the children and recipients played a key role. Across age groups, this study of preschool children (total *N* = 137) demonstrates a degree of effect of self-relevance on preschool children’s sharing in first-party and third-party situations, with highly self-relevant recipients receiving a more preferential share in the dictator game than those with low self-relevance, although this effect was stronger in the older preschool children.

## Introduction

Sharing behavior is a vital research topic in the field of children’s moral and developmental psychology. Sharing resources with others is an important prosocial behavior (Markovits et al., 2003; Steinbeis and Over, 2017). Recently, many studies have focused on sharing behavior in early childhood (Rochat et al., 2009; Svetlova et al., 2010; Baumard et al., 2012; Paulus et al., 2015). The growing focus of these studies was mainly on the many factors that influence sharing behavior and decisions in young children, including recipients, distributors, objects and situations related to sharing resources (Kanngiesser and Warneken, 2012; Crittenden and Zes, 2015; Kogut et al., 2015; Gasiorowska et al., 2016; Hao et al., 2016; Malti et al., 2016). Rogers first discovered the self-reference effect, he thought the memory effect of the self-associated memory material was significantly better than that of other coding conditions, namely the self-referential effect (Rogers et al., 1977). Symons and Johnson believe that the self-reference effect occurred because the ego was a well-developed and frequently used structure (Symons and Johnson, 1997), which facilitated the fine processing of information (Brown et al., 1986) and tissue processing (Klein et al., 1994). The generation of self-referential effects was related to the degree of development of self-concepts.

A study by Sui and Zhu (2005) found that 5-year-old children already exhibit self-reference effects (Sui and Zhu, 2005). Zhou further found that 4-year-old children already have a self-reference effect (Zhou et al., 2010). Later, some researchers found that 3-year-old children already have a self-reference effect (Mei Haibo, 2013). The above research showed that the self-concept of 3–6 age year children was fully developed, which could not only distinguish themselves from others, but also could produce memory self-referencing processing effects according to this familiarity.

Researchers often described the level of familiarity and association between individuals and others using self-relevance (Farb et al., 2007; Zhou et al., 2017). Researchers often used the IOS scale to measure the degree of self-relevance between individuals and others. Those who scored 5–7 points to be highly self-related (such as close friends), and those who scored 3–4 points were moderately self-related (such as acquaintances), those who will score at 1–2 points for others as low self-related (such as strangers) (Aron et al., 1992; Zhong et al., 2015).

Studies (Fehr et al., 2008; Moore, 2009) have provided evidence that preschool children already react differently to different kinds of recipients in resource allocation tasks and that they are very sensitive to the principle of reciprocity in social interaction and communication (Kenward et al., 2015; Paulus et al., 2015; Lu and Chang, 2016; Mulvey et al., 2016; Paulus, 2016b; Xiong et al., 2016; Scharpf et al., 2017). In resource allocation tasks, children share more generously with in-group members or recipients with similar interests than with out-group members (Sparks et al., 2017). Studies from some western cultures have shown that preschool children tend to share more resources with friends than with mere acquaintances (Costin and Jones, 1992; Rao and Stewart, 1999; Paulus and Moore, 2014), or they share more with friends than with disliked peers, non-friends or strangers (Birch and Billman, 1986; Fehr et al., 2008; Olson and Spelke, 2008). However, recent studies (Scharpf et al., 2016, 2017; Cowell et al., 2017) pointed to cross-cultural differences in young children’s sharing. A study from eastern Africa (Scharpf et al., 2017) points out that sharing among young children in Uganda did not depend on the social relationship between the sharer and the recipient. One study (Rochat et al., 2009) indicated that children from a collectivist culture are more likely to share goods with others.

The social relationships from previous theories (Newcomb and Bagwell, 1995) were divided into kin, friends, acquaintances, strangers and disliked peers or enemies. Friends are defined as people with close, interpersonal ties and positive, amiable preexisting relationships (Jehn and Shah, 1997). Acquaintances, strangers, and disliked peers or enemies belong to the group of non-friends. Acquaintances are defined as people with limited familiarity, intimacy and contact. Strangers are defined as people with hardly any familiarity or common experience. Enemies or disliked peers are defined as people with a few common experiences and negative or even contemptuous relationships. However, acquaintances, strangers, and disliked peers or enemies were not strictly distinguished in many previous studies about early sharing. For example, acquaintances were mixed with disliked peers in one study (Moore, 2009), which defined peers who dislike one another yet play together as acquaintances. Additionally, in a number of studies (Birch and Billman, 1986; Costin and Jones, 1992; Olson and Spelke, 2008; Cowell et al., 2017), acquaintances were recipients in resource sharing tasks, but the familiarity and intimacy between participants and recipients were not strictly and systematically manipulated (Blake et al., 2015). Therefore, these studies only compared two levels of relationships between participants and recipients, preexisting and non-existing, such as friends and strangers, and positive and negative relationships, such as friends and disliked peers.

According to the self-relevance concept from China, interpersonal relationships present differences depending on whether a subject is close to oneself or distant from oneself thus; self-relevance should be manipulated to different degrees, such as high self-relevance, moderate self-relevance and low self-relevance (Fan et al., 2013; Tan et al., 2015). One study (Zhong et al., 2015) from China supposed that self-relevance influences prosocial behavior among adults and found that the higher the degree of overlap was between oneself and others, the more obvious the presence of helping behavior. What about the impact of self-relevance on prosocial behavior in preschool children? This study will systemically manipulate self-relevance based on the familiarity and intimacy between distributors and recipients as well as explore the differences when children make sharing decisions about recipients with different degrees of self-relevance, such as friends, acquaintances, and strangers.

In addition to the relationship between recipients and distributors, the situations of resource allocation were thought to affect sharing behaviors among young children. In previous studies, situations based on different payoff structures were mainly divided into non-costly, costly, and envy situations. Studies indicated that children aged 2 years old start to display a strong tendency to share with others (especially with intimate peers) in non-costly situations (Brownell et al., 2009) but that children aged 3–6 exhibit a reduction in sharing and are not willing to sacrifice their own interests when in costly situations (Fehr et al., 2008; Smith et al., 2013). Similarly, children aged 3–8 often refuse to deprive themselves of their own dominant position to choose the option of benefiting others. Researchers (Brownell et al., 2009) found that children dislike others who get more than them (motivated by social comparison); thus, children are less willing to share with others in envy situations (Shaw et al., 2014; Sheskin et al., 2014; Williams et al., 2014). For example, 5-year-old children will refuse the option of two resources for each child to choose the option of one resource going to themselves and others getting nothing. Therefore, these studies indicate that young children aged 3–6 are very sensitive to situations that affect their payoff. One study (Kanngiesser and Warneken, 2012) indicated that children in early childhood have an ability to take merit into account in third-party situations but that merit-based sharing in first-party situations does not appear until school age. Do young children take self-relevance into account in sharing? Additionally, is there any difference between first-party and third-party situations? Most studies (Rochat et al., 2009; Paulus et al., 2013) demand that young children share a resource between themselves and others in first-person scenarios and rarely exclude the self-interests of participants. Therefore, it remains an open question whether children will behave in accordance with self-relevance when sharing resources in first-person and third-person situations.

## Experiment 1 the Impact of Self-Relevance on Preschool Children’s Sharing in First-Party Situations

### Aim and Hypothesis

Aim: Experiment 1 requires participants to share resources between themselves and others and participants also as resource recipients. The study allowed participants to choose “sharing” or “non-sharing” based on three different conditions (non-costly, costly, and envy). The study records the number of times the participants choose to “sharing” with others, and explore the role of self-relevance in participants’ sharing behavior.

Hypothesis: Compared with acquaintances and strangers, the participants shared more behaviors with close friends; compared with strangers, the participants shared more behaviors with acquaintances; compared with 3–4 years old children, 5–6 years old children will share more behaviors, which means that the degree of self-relevance in the 5–6 years old children was more stable.

### Research Method

#### Participants

The participants in Experiment 1 consisted of 75 children aged 3–6 years from an urban kindergarten located in China. The participants were divided into two groups based on age: 3–4 years (*n* = 39, 21 males and 15 females; *M* = 53.00 months, *SD* = 5.09, range = 38–58) and 5–6 years (*n* = 36, 15 males and 21 females; *M* = 68.19 months, *SD* = 5.44, range = 60–77). No children were suffering from mental or neurological disorders, and all spoke Chinese as their first language. This study was approved by the ethics committee of Hunan Normal University. Informed written consent was obtained from the parents of all participants. The participants received gifts.

#### Materials

##### Experiment design

This experiment used a two-factor mixed design of 3 (self-correlation: high self-related – friends, medium self-related – acquaintance, low self-related – stranger) × self 2 (age group: 3–4 years old, 5–6 years old group). Self-relevance is the intra-group variable, including high, medium, and low levels; the age group is the inter-group variable, including the 3–4-year-old group and the 5–6-year-old group. The dependent variable is the number of times the participants chooses to share generously with the recipient.

##### Photos of recipients

Photos of friends, acquaintances and strangers served as three different self-relevance recipients, the friends, acquaintances, and strangers selected in this study had the same age as the participants. All photos were standardized at a size of 2.5 × 3.5 cm, containing an image of the head with a neutral facial expression. The genders of the three recipients were matched.

##### IOS scale

The degrees of self-relevance between oneself and others were measured using the IOS scale. The people whose scores ranged from 5 to 7 were highly self-relevant others (e.g., friends), those whose scores ranged from 3 to 4 were moderately self-relevant others (e.g., acquaintances), and these whose scores ranged from 1 to 2 were lowly self-relevant others (e.g., strangers) (Aron et al., 1992; Zhong et al., 2015).

##### Food in sharing task

Researchers chose the children’s favorite food, M&M chocolates, as the sharing resource. Children were required to rate their preference for the food using a cartoon expression with one of three associate point values (like, neither like nor dislike, dislike) before the experiment (Birch and Billman, 1986; Crittenden and Zes, 2015).

#### Procedure

##### Food preference ratings

The experiment was conducted in a quiet room located in the kindergarten. The experimenter provided a sample of food (M&M’s, M&M is a chocolate bean in the United States, in 2004, M&M’s was named the most favorite food in the United States) to the children. After tasting, the children chose a cartoon expression (like, neither like nor dislike, dislike) to rate the food. If a child reported that they liked the food, the child would continue with the experiment as a participant.

##### Recipients’ selection

First, photos of all classmates were provided, with a corresponding name written on the back of each photo. The participants were asked to find their own photo and divide the other photos into three categories: like, neither like nor dislike, and dislike. Second, the participants chose their three favorite photos from the “like” category, one of which had a score of 5–7 on the IOS scale, indicating high self-relevance, and was identified as a close friend. Third, the experimenters randomly chose three photos from the “neither like nor dislike” category, one of which had a score of 3–4, indicating moderate self-relevance, and was identified as a regular acquaintance. Finally, the experimenters provided three photos of strangers, one of which had a score (Zhong et al., 2015). In addition, when the experimenters selected photos, they made sure that the three recipients (close friends, acquaintances, and strangers) were the same gender.

##### Resource sharing task

**FIGURE 1:**
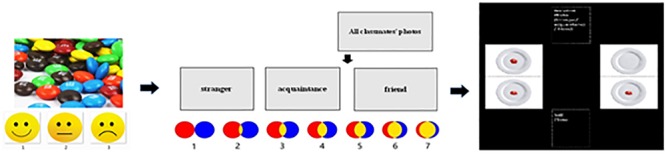
Experimental process.

After the completion of the two procedures above, the participants sat on a chair facing a desk. The experimenters introduced the resource sharing task to the participant and told him/her how to share chocolates with the recipients (close friends, acquaintances, and strangers). The experimenters put the photo of the participant before him and one of recipients’ photos opposite him on the desk and then displayed two options (as shown in Figure 1). During the experiment, the experimenters not only stated the experimental content but also assisted with the physical operation to help participants understand it. After children decided, the experimenters would record the selected options.

Experiment 1 presented 3 blocks to participants (3–4-year-olds and 5–6-year-olds), which consisted of 9 trials in each block, amounting to 27 trials in total. Each block contained three different trial types according participants’ payoff: non-costly, costly, and envy situations, non-costly situations. The experimenter asked the participants to choose between two options [the participant and the recipient, respectively, have a sugar (1/1), the other is the participants have a sugar and the recipient have not a sugar (1/0)], costly situations [the experimenter asked the participants to choose between two options. One option was that the participants and the recipient, respectively, have one sugar (1/1), and the other option was that the participants had two sugars while others had no sugar (2/0)], envy situations [he experimenter asked the participants to make a choice between the two options. One option was that the participant and the recipient did not have sugar (0/0), the other option was that the participant had one sugar and the recipient had two sugars (1/2)]. In each trial, the participants made decisions between two options by using forced-choice resource sharing to form a simple dictator game. The dictator game is a very simple, one-shot decision situation in which the dictator can distribute resources to recipients in any way, and the recipient has to accept the allocation (Gummerum et al., 2008; Moore, 2009; Wilkening, 2009). The orders of the trials and options were balanced between the blocks and participants.

#### Data Analysis

The experimenter scored the results of the participant selection (Paulus, 2016a). If the participant chooses to share generously with the recipient (friend, acquaintance, stranger) and benefit from others, then the participant will get 1 point in this trial. For example, for costly conditions and non-costly conditions, the participant selects the option (1/1), scores 1 point; the envy condition, the participants select the option (1/2), scores 1 point. If the participant chooses another option and record 0 points. The scores obtained by all the trials of all blocks are accumulated according to the trial type and are regarded as the share scores of the accepted objects.

Experiment 1 mainly investigated the influence of self-relevance on children’s sharing behaviors and, specifically, whether higher self-relevance between recipients and participants made it more likely that children would share resources with the recipients. Therefore, this experiment adopts a repeated measure analysis of variance (ANOVA) using a mixed model with two factors, 3 self-relevance (high self-relevance – friends, moderate self-relevance – acquaintances, and low self-relevance – strangers) × 2 age (the 3–4-year-old group and the 5–6-year-old group). The degrees of freedom of the F-ratio were corrected according to the Greenhouse–Geisser method.

### Results

#### Self-Relevance Ratings

The post-experiment assessment using an IOS scale showed a significant main effect of self-relevance in the recipients, *F*(2,72) = 1381.54, *p* < 0.001, $\eta_{p}^{2}$ = 0.95; *post hoc* testing revealed the self-relevance scores of friends’ recipients to be significantly higher than those of acquaintances recipients and strangers’ recipients and the self-relevance scores of acquaintances recipients to be higher than those of strangers’ recipients, *p* < 0.001.

#### Sharing Behavior in Non-costly Situations

Under non-costly conditions, a multiple ANOVA showed a highly significant main effect of self-relevance, *F*(2,146) = 30.96, *p* < 0.001, $\eta_{p}^{2}$ = 0.30; *post hoc* multiple comparison revealed that the sharing scores of friends’ recipients were significantly higher than those of acquaintances recipients and strangers and that the sharing scores of acquaintances recipients were significantly higher than those of strangers, *p* < 0.05. The main effect of age group was not significant, *F*(1,73) = 0.08, *p* > 0.05, $\eta_{p}^{2}$ = 0.01. There were no significant interactions between age and self-relevance, *F*(2,146) = 2.30, *p* > 0.05, $\eta_{p}^{2}$ = 0.03.

For the data of the 3–4-year-old participants, the one-factor ANOVA of self-relevance showed a highly significant main effect of self-relevance, *F*(2,114) = 10.49, *p* < 0.001, $\eta_{p}^{2}$ = 0.22. *Post hoc* multiple comparison revealed that the sharing scores of friends’ recipients were significantly higher than those of acquaintances recipients (*p* < 0.01) and strangers’ recipients (*p* < 0.001), and there was no significant difference in the sharing scores of moderately self-relevant recipients and lowly self-relevant recipients (*p* > 0.05).

**FIGURE 2:**
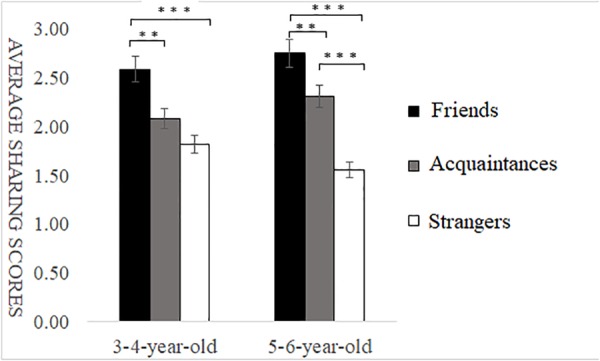
Sharing scores of different types of self-relevant recipients in non-costly situations. ^∗^*p* < 0.05, ^∗∗^*p* < 0.01, ^∗∗∗^*p* < 0.001.

For the data of the 5–6-year-old participants, the one-factor ANOVA of self-relevance showed a highly significant main effect of self-relevance, *F*(2,115) = 21.99, *p* < 0.001, $\eta_{p}^{2}$ = 0.39. *Post hoc* multiple comparison revealed that the sharing scores of friends’ recipients were significantly higher than those of acquaintances recipients (*p* < 0.01) and strangers’ recipients (*p* < 0.001), and the sharing scores of acquaintances recipients were significantly higher than those of strangers’ recipients (*p* < 0.001) (as shown in Figure 2).

#### Sharing Behavior in Costly Situations

**FIGURE 3:**
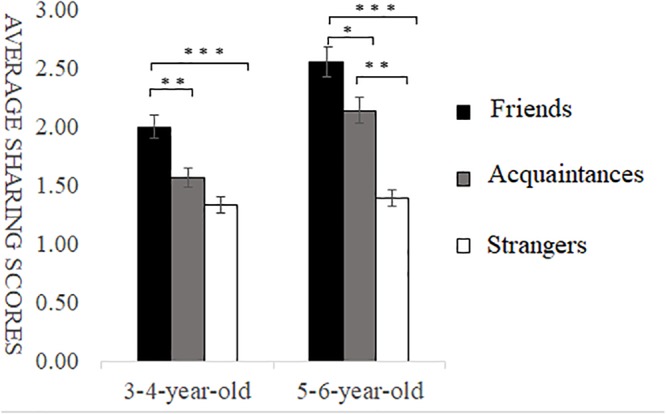
Sharing scores of different types of self-relevant recipients in costly situations. ^∗^*p* < 0.05, ^∗∗^*p* < 0.01, ^∗∗∗^*p* < 0.001.

Under costly conditions, a multiple ANOVA showed a highly significant main effect of self-relevance, *F*(2,146) = 26.17, *p* < 0.001, $\eta_{p}^{2}$ = 0.26. *Post hoc* multiple comparison revealed that the sharing scores of friends’ recipients were significantly higher than those of acquaintances recipients and strangers’ recipients, and the sharing scores of acquaintances recipients were significantly higher than those of strangers’ recipients, *p* < 0.001. The main effect of age was marginally significant, with children aged 5–6 sharing more than those aged 3–4, *F*(1,146) = 3.68, *p* = 0.06, $\eta_{p}^{2}$ = 0.05. There was also a marginally significant interaction between self-relevance and age, *F*(2,146) = 2.70, *p* = 0.07, $\eta_{p}^{2}$ = 0.04. Simple effects analysis showed that, for participants aged 3–4, the sharing scores of friends’ recipients were significantly higher than those of acquaintances recipients (*p* < 0.01) and strangers’ recipients (*p* < 0.01), and the sharing scores of acquaintances recipients were not significantly different than those of strangers’ recipients (*p* > 0.05). For participants aged 5–6, the sharing scores of friends’ recipients were significantly higher than those of acquaintances recipients (*p* < 0.05) and strangers’ recipients (*p* < 0.001), and the sharing scores of acquaintances recipients were significantly higher than those of strangers’ recipients (*p* < 0.01) (as shown in Figure 3).

#### Sharing Behavior in the Envy Situation

**FIGURE 4:**
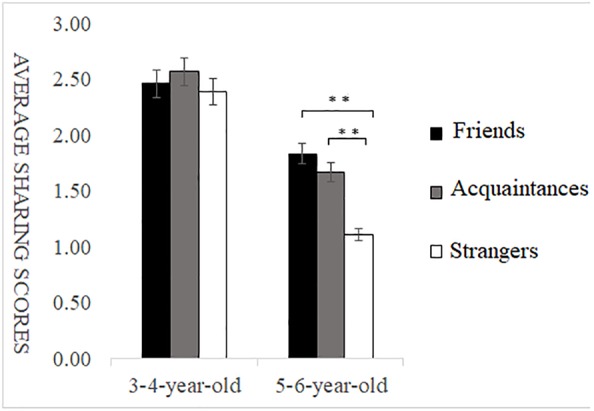
Sharing scores of different types of self-relevant recipients in envy situations. ^∗^*p* < 0.05, ^∗∗^*p* < 0.01, ^∗∗∗^*p* < 0.001.

Under envy conditions, a multiple ANOVA showed a highly significant main effect of self-relevance, *F*(2,146) = 8.82, *p* < 0.01, $\eta_{p}^{2}$ = 0.11. *Post hoc* multiple comparison revealed that the sharing scores of friends’ recipients were significantly higher than those of acquaintances recipients and strangers’ recipients, and the sharing scores of acquaintances recipients were significantly higher than those of strangers’ recipients, *p* < 0.01. The main effect of age was highly significant, with children aged 3–4 sharing more than those aged 5–6, *F*(1,146) = 16.52, *p* < 0.001, $\eta_{p}^{2}$ = 0.19. There was also a significant interaction between self-relevance and age, *F*(2,146) = 4.70, *p* < 0.05, $\eta_{p}^{2}$ = 0.06. Simple effects analysis showed that, for participants aged 3–4, sharing scores were not significantly different among friends’ recipients, acquaintances recipients and strangers’ recipients (*p* > 0.05). For participants aged 5–6, the sharing scores of friends and acquaintances recipients were significantly higher than those of strangers’ recipients (*p* < 0.01), and the sharing scores of friends’ recipients were not significantly different than those of acquaintances recipients (*p* > 0.05) (as shown in Figure 4).

### Discussion

Before the resource allocation task, the results of the self-relevance rating between participants and recipients using an IOS scale showed that the scores of self-relevance were extremely significantly different since the scores of highly self-relevant recipients were significantly higher than those of moderately self-relevant recipients, and the scores of moderately self-relevant recipients were significantly higher than those of lowly self-relevant recipients. These results indicate that participants were able to distinguish the different degree of self-relevance among recipients and prove the validity of manipulating self-relevance in the experiment.

In Experiment 1, the results in non-costly situations showed that preschool children shared more with highly self-relevant recipients than moderately self-relevant recipients as well as more with moderately self-relevant recipients than with lowly self-relevant recipients, which indicated that preschool children could not only distinguish the different degree of self-relevance among recipients but also demonstrate different sharing behavior and decisions based on this distinction. Preschool children are able to take self-relevance into account when sharing resources with others. The experimental results bear our hypothesis in the experiment. These results were consistent with those reported in previous research. Previous studies found that children are more likely to share with friends and acquaintances (Birch and Billman, 1986; Fehr et al., 2008; Olson and Spelke, 2008) and that children share more with friends than acquaintances (Costin and Jones, 1992; Olson and Spelke, 2008). These studies showed that early sharing behavior is obviously influenced by the closeness and intimacy of relationships between oneself and recipients and that parochialism plays an important role in early sharing behaviors (Bernhard et al., 2006).

However, the results under non-costly conditions did not reveal a significant difference between the age groups of 3–4 and 5–6. Many studies (Brownell et al., 2009) about early sharing behavior often examined children’s sharing behavior in non-costly situations. In resource distribution tasks without a sacrifice of self-interest, young children exhibited a strong tendency for sharing (Olson and Spelke, 2008; Kenward and Dahl, 2011; Baumard et al., 2012). For example, at the age of two, young children have shown a strong willingness to share with others in non-costly situations (Brownell et al., 2009). These studies indicated that children are more willing to share under non-costly conditions. Therefore, participants in non-costly situations tended to share resources at the ages of 3–4 and 5–6, and there was no difference between the two age groups.

Experiment 1 under costly conditions also showed that, when children shared resources with others, the scores of highly self-relevant recipients were significantly higher than those of moderately self-relevant recipients, and the scores of moderately self-relevant recipients were significantly higher than those of lowly self-relevant recipients. These results indicated that, even in situations where participants need to sacrifice self-interest, children still perform differently in sharing behaviors and decisions based on the self-relevance between participants and recipients. The experimental results bear our hypothesis in the experiment. This result is similar to the results in non-costly situations, and the effect of self-relevance on children’s sharing behaviors is observed. Unlike in non-costly conditions, the effect of age difference on sharing behaviors was significant in costly situations, with the scores of 3–4-year-old children being significantly lower than those of 5–6-year-old children. This result was consistent with those of previous studies. Other researchers (Thompson et al., 1997) pointed out that, when self-interests are decreased, children’s willingness to share is weakened. In costly situations, children will start to occasionally share goods that they gained with others when they are 3 years old. Previous researchers (Ugurel-Semin, 1952) pointed out that children could make the leap to generously sharing with others at the age of 5 or 6. Thus, the loss of self-interest led to the decline of the sharing tendency in children aged 3–4, but children aged 5–6 were still willing to generously share with others. In addition, the results in costly situations showed that children aged 3–4 were able to distinguish highly self-relevant recipients from moderately self-relevant recipients and lowly self-relevant recipients but were unable to distinguish moderately self-relevant recipients from lowly self-relevant recipients; however, children aged 5–6 were able to clearly distinguish among the three different kinds of self-relevant recipients. These results suggest that preschool children could be able to take self-relevance into account when performing sharing behaviors in costly situations. However, in costly situations, children aged 5–6 showed a stronger and steadier degree of effect on sharing behaviors and decisions than did children aged 3–4.

The results of Experiment 1 under envy conditions also showed that scores of highly self-relevant recipients were significantly higher than those of moderately self-relevant recipients, and the scores of moderately self-relevant recipients were significantly higher than those of lowly self-relevant recipients. Thus, even in cases where self-interest advantages were threatened, children still performed differently in sharing behaviors and decisions based on the self-relevance between participants and recipients. The experimental results bear our hypothesis in the experiment. Interestingly, the results in envy situations indicate that there was a significant difference based on age in the sharing behaviors among preschool children, with children aged 5–6 years sharing significantly less than those aged 3–4. Jealousy is a kind of negative feeling experienced by individuals; when individuals realize others have an advantage that they lack, they display mixed feelings of inferiority, hostility, and resentment (Hart and Behrens, 2013; Mize et al., 2014). Recently, research (Sheskin et al., 2016) pointed out that, when their own resource advantage was threatened, children’s sharing behaviors developed very slowly. Children compare themselves to others, and when, they realize others have more resources than them, jealousy is induced (Shaw and Olson, 2012). Awareness of competition will decrease prosocial and sharing behaviors in preschool children (Pappert et al., 2016). In this case, children tend to choose the options favoring themselves and accept the unfair advantage (Blake and Mcauliffe, 2011; Lobue et al., 2011).

In envy conditions, recipients in sharing tasks were peers, which induced strong feelings of envy in the children aged 5–6. Early social comparison emotions, including envy, reduced early prosocial motivation (Steinbeis and Singer, 2013). Fehr and Schmidt (1999) pointed out that, when the participants’ income was lower than others, they experienced envy. Therefore, in envy conditions, the sharing behaviors of children aged 5–6 were significantly reduced under feelings of intense jealousy. Even so, the self-relevance of recipients was still considered when children aged 5–6 shared resources. This result indicated that, in envy conditions, children aged 5–6 would make a sharing decision according to self-relevance, and the effect of the degree of self-relevance still exists. However, there was no significant difference among children aged 3–4 in sharing behaviors, and the effect of the degree of self-relevance disappeared. On the one hand, this may be due to the immaturity of self-consciousness and self-relevance among children aged 3–4 (Gerardi-Caulton, 2000). The participants could not distinguish among the three types of self-relevant recipients very well because of their envy. On the other hand, it may be the limitations of recognition (Paulus et al., 2015) that make it so that children aged 3–4 could not distinguish situations that elicited jealousy (Masciuch and Kienapple, 1993). In envy conditions, children aged 3–4 failed to compare themselves with others who faced different payoffs. Thus, the lack of envy generated by social comparison made no difference among these recipients.

The results of Experiment 1 proved that children are more generous to highly self-relevant recipients, regardless of whether in non-costly conditions, costly conditions or, even, envy conditions. This finding suggests that young children are able to distinguish between different self-relevant recipients and discriminate differently in sharing behaviors. This is the effect of the degree of self-relevance on children’s sharing behavior. Of course, the effect of self-relevance is steadier in children aged 5–6 than in children aged 3–4.

Experiment 1 investigated the tendency of children to share with different self-relevant recipients when they allocate resources between themselves and recipients. Previous studies (Benenson et al., 2007; Blake and Rand, 2010; Gummerum et al., 2010) have shown that children’s sharing behaviors are inevitably disturbed when their own interests are involved, although children exhibit a strong tendency of sharing from an early age. To eliminate the interference of self-interests, participants were asked to allocate resources between two recipients, and participants are dictators but not recipients in Experiment 2. In third-party situations, will children share resources with others according to the self-relevance of recipients?

## Experiment 2: the Impact of Self-Relevance on Preschool Children’s Sharing in Third-Party Situations

### Aim and Hypothesis

Aim: Experiment 2 requires participants to allocate resources to other people (close friends, acquaintances, and strangers) as distributors and participants no accepted resources. According to the resource allocation paradigm, the study sated three task scenarios (no bias, high self-bias, low self-bias) and each task scenarios provides two distribution options (multiple sharing, less sharing). Last, the study records participants’ choice of “multiple sharing” and explore the role of self-relevance in children’s sharing behavior.

Hypothesis: Compared with acquaintances and strangers, the participants shared more behaviors with close friends; compared with strangers, the participants shared more behaviors with acquaintances; compared with 3–4 years old children, 5–6 years old children will share more behaviors, which means that the degree of self-relevance in the 5–6 years old children was more stable.

### Research Method

#### Participants

The participants in Experiment 2 consisted of 62 children aged 3–6 years from a kindergarten located in urban China. The participants were divided into two groups based on age: 3–4 years (*n* = 30, 15 males and 15 females; *M* = 52.50 months, *SD* = 3.34, range = 46–59) and 5–6 years (*n* = 32, 16 males and 16 females; *M* = 65. 97 months, *SD* = 2.95, range = 61–71). No children suffered from mental or neurological disorders, and all spoke Chinese as their first language. This study was approved by the ethics committee of Hunan Normal University. Informed written consent was obtained from the parents of all the participants. The participants received gifts.

#### Experimental Materials

Same as Experiment 1.

#### Procedure

(1) Food preference ratings (same as Experiment 1).
(2) Recipients’ selection (same as Experiment 1).
(3) Resource sharing task (similar to Experiment 1).

The difference between Experiments 1 and 2 at this step is that participants need to allocate chocolates to two recipients (close friends, acquaintances, and strangers), but participants themselves are not recipients. Experiment 2 presented 3 blocks of 27 trials in total in the same manner as Experiment 1. Each block contained three different trial types according to participants’ payoffs: non-bias, high self-bias, and low self-bias. For example, participants made the decision between a 3/1 option and a 1/3 option in non-bias trials (under the non-bias condition, there were two options for the participant, one option 3/1 was to give the friend three sugars and give the acquaintance a sugar, the other option 1/3 was to give the friend a sugar and give the acquaintance three sugars), between a 3/1 option and a 2/2 option in high self-bias trials (under the high self-bias condition, the participant had two options, one option 3/1 was given friends three sugars and give acquaintances one sugar; the other option 2/2 was, respectively, giving friends and acquaintances two sugars), between 1/3 and 2/2 options in low self-bias trials (under the low self-bias condition, the participant had two options, one option 1/3 was given friends one sugars and give acquaintances three sugar; the other option 2/2 was, respectively, given friends and acquaintances two sugars). In each trial, participants allocated resources to a higher self-relevant recipient and a lower self-relevant recipient. The orders of trials and options were balanced between the blocks and participants.

#### Data Analysis

The experimenter recorded and coded the participant’s decision into sharing scores (Paulus, 2016a). When participants chose an option to benefit one of the recipients, the corresponding recipient would get a score of one. For example, if participants chose to share more (3/1) instead of sharing less (1/3) with higher self-relevant recipients in non-bias trials, then the higher self-relevant recipients would get a score of one. Scores in all trials were calculated according to the trial type, which was considered the sharing scores of the corresponding recipients.

This experiment adopted a repeated measures analyses of variance (ANOVA) using a mixed model with two factors, 3 self-correlations (high self-related – friends, medium self-related – acquaintances, low self-related – strangers) × 2 (age: 3 to 4 years old group, 5 to 6 years old group). The degrees of freedom of the F-ratio were corrected according to the Greenhouse–Geisser method.

### Results

#### Self-Relevance Ratings

The post-experiment assessment using an IOS scale showed a significant main effect of the type of self-relevance in the recipients, *F*(2,59) = 1056.91, *p* < 0.001, $\eta_{p}^{2}$ = 0.95. *Post hoc* testing revealed the self-relevance scores of friends’ recipients to be significantly higher than those of acquaintances’ recipients and strangers’ recipients, and the self-relevance scores of moderately self-relevant recipients were higher than those of strangers’ recipients, *p* < 0.001.

#### Sharing Behavior in Non-bias Situations

Under non-bias conditions, a multiple ANOVA showed a highly significant main effect of self-relevance, *F*(2,120) = 57.95, *p* < 0.001, $\eta_{p}^{2}$ = 0.49. *Post hoc* multiple comparison revealed that the sharing scores of friends’ recipients were significantly higher than those of acquaintances’ recipients and strangers’ recipients, and the scores of acquaintances’ recipients were significantly higher than those of strangers’ recipients, *p* < 0.001. The main effect of age was not significant, *F*(1,60) = 1.07, *p* > 0.05, $\eta_{p}^{2}$ = 0.02. There were no significant interactions between age and self-relevance, *F*(2,120) = 0.02, *p* > 0.05, $\eta_{p}^{2}$ = 0.001.

For the data of the 3–4-year-old participants, the one-factor ANOVA of self-relevance showed a highly significant main effect of self-relevance, *F*(2,87) = 31.53, *p* < 0.001, $\eta_{p}^{2}$ = 0.52. *Post hoc* multiple comparison revealed that the sharing scores of friends’ recipients were significantly higher than those of acquaintances recipients and strangers’ recipients, and the scores of acquaintances recipients were higher than those that of strangers’ recipients (*p* < 0.001).

**FIGURE 5:**
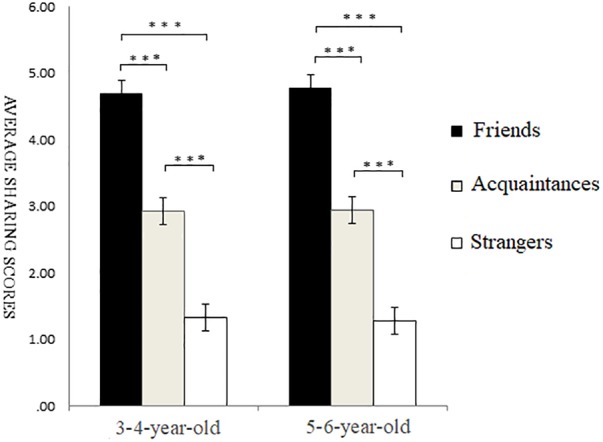
Sharing scores of different types of self-relevant recipients in non-bias situations. ^∗^*p* < 0.05, ^∗∗^*p* < 0.01, ^∗∗∗^*p* < 0.001.

For the data of the 5–6-year-old participants, the one-factor ANOVA of self-relevance showed a highly significant main effect of self-relevance, *F*(2,93) = 27.42, *p* < 0.001, $\eta_{p}^{2}$ = 0.47. *Post hoc* multiple comparison revealed that the sharing scores of friends’ recipients were significantly higher than those of acquaintances recipients and strangers’ recipients (*p* < 0.001), and the scores of acquaintances recipients were significantly higher than those of strangers’ recipients (*p* < 0.01) (as shown in Figure 5).

#### Sharing Behavior in High Self-Bias Situations

In high self-bias conditions, a multiple ANOVA showed a significant main effect of self-relevance, *F*(2,120) = 33.88, *p* < 0.001, $\eta_{p}^{2}$ = 0.36. *Post hoc* multiple comparison revealed that the sharing scores of friends’ recipients were significantly higher than those of acquaintances recipients and strangers’ recipients, and the scores of acquaintances recipients were significantly higher than those of strangers’ recipients, *p* < 0.001. The main effect of age was not significant, *F*(1,60) = 1.07, *p* > 0.05, $\eta_{p}^{2}$ = 0.02. There were no significant interactions between age and self-relevance, *F*(2,120) = 1.60, *p* > 0.05, $\eta_{p}^{2}$ = 0.03.

For the data of the 3–4-year-old participants, the one-factor ANOVA of self-relevance showed a highly significant main effect of self-relevance, *F*(2,87) = 10.83, *p* < 0.001, $\eta_{p}^{2}$ = 0.27. *Post hoc* multiple comparison revealed that the sharing scores of friends’ recipients (*p* < 0.001) and acquaintances recipients (*p* < 0.01) were significantly higher than those of strangers’ recipients and that there was no significant difference between highly self-relevant recipients and acquaintances recipients (*p* > 0.05).

**FIGURE 6:**
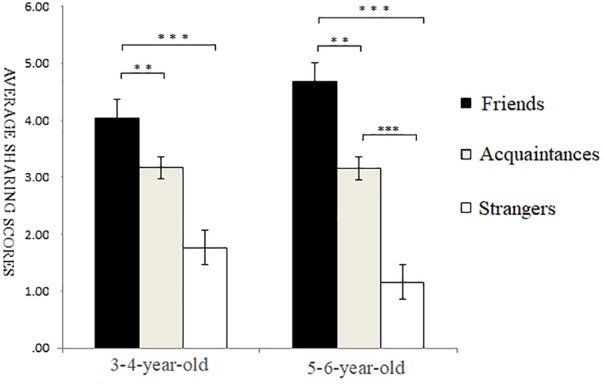
Sharing scores of different types of self-relevant recipients in high self-bias situations. ^∗^*p* < 0.05, ^∗∗^*p* < 0.01, ^∗∗∗^*p* < 0.001.

For the data of the 5–6-year-old participants, the one-factor ANOVA of self-relevance showed a highly significant main effect of self-relevance, *F*(2,93) = 24.37, *p* < 0.001, $\eta_{p}^{2}$ = 0.44. *Post hoc* multiple comparison revealed that the sharing scores of friends’ recipients were significantly higher than those of acquaintances recipients (*p* < 0.01) and strangers’ recipients (*p* < 0.001), and the scores of acquaintances recipients were significantly higher than those of strangers’ recipients (*p* < 0.001) (as shown in Figure 6).

#### Sharing Behaviors in Low Self-Bias Situations

In low self-bias conditions, a repeated measures ANOVA showed a highly significant main effect of self-relevance, *F*(2,120) = 26.72, *p* < 0.001, $\eta_{p}^{2}$ = 0.32. *Post hoc* multiple comparison revealed that the sharing scores of friends’ recipients and acquaintances recipients were significantly higher than those of strangers’ recipients (*p* < 0.05), and there was no significant difference between friends’ recipients and acquaintances recipients (*p* > 0.05). The effect of age was not significant, *F*(1,60) = 0.001, *p* > 0.05, $\eta_{p}^{2}$ = 0.001.

**FIGURE 7:**
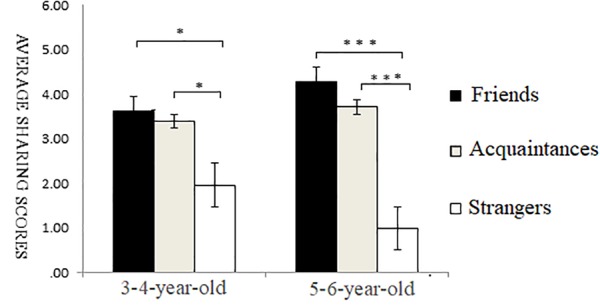
Sharing scores of different types of self-relevant recipients in low self-bias situations. ^∗^*p* < 0.05, ^∗∗^*p* < 0.01, ^∗∗∗^*p* < 0.001.

There was also a marginally significant interaction between self-relevance and age, *F*(2,120) = 2.76, *p* = 0.06, $\eta_{p}^{2}$ = 0.05. Simple effects analysis showed that, for participants aged 3–4, the sharing scores of friends recipients and acquaintances recipients were significantly higher than those of strangers’ recipients (*p* < 0.05), and there was no significant difference between friends recipients and acquaintances recipients (*p* > 0.05); for participants aged 5–6, the sharing scores of friends recipients and acquaintances recipients were significantly higher than those of strangers’ recipients (*p* < 0.001), and there was not significant difference between friends recipients and acquaintances recipients (*p* > 0.05) (as shown in Figure 7).

### Discussion

To eliminate the interference of self-interest, participants were distributors but not recipients of resources in Experiment 2. The results in non-bias conditions showed that children aged 3–4 and aged 5–6 both clearly distinguished among highly self-relevant, moderately self-relevant and lowly self-relevant recipients, and effects of the degree of self-relevance appeared in preschool children’s sharing behaviors, which is in accordance with the experimental hypothesis. Some studies point out that children share more generously with in-group recipients than with out-group members (Olson and Spelke, 2008; Moore, 2009; Sparks et al., 2017). Usually, highly self-relevant recipients are closer to oneself and more likely to belong to in-group recipients. Lowly self-relevant recipients are more likely to belong to out-group members. When choosing between options without any bias, children will choose to share more resources with those recipients who have higher self-relevance.

The results from the high-self bias situations show that children clearly distinguished between highly self-relevant, moderately self-relevant, and lowly self-relevant recipients, and the effect of the degree of self-relevance appeared in preschool children’s sharing behaviors, which is in accordance with the experimental hypothesis and the results in the non-bias conditions. However, the results of the data of participants aged 3–4 showed that they could not distinguish between more highly self-relevant recipients and moderately self-relevant recipients, but the results of the data of participants aged 5–6 showed that they could distinguish among all three types of self-relevant recipients. This result indicated that children aged 5–6 could better share resources according to the self-relevance of recipients than children aged 3–4. The effect of the degree of relevance became steadier as the children developed.

The results in low self-bias conditions found that children aged 3–4 and children aged 5–6 could distinguish the highly self-relevant recipients from the lowly self-relevant recipients and distinguish the moderately self-relevant recipients from the lowly self-relevant recipients. However, neither could distinguish the highly self-relevant recipients from the moderately self-relevant recipients. In low self-bias conditions, one option provides more resources to the lower self-relevant recipients. This tendency to provide an advantage of resources to the lower self-relevant recipients may betray children’s psychological preference to in-group recipients (Bauer et al., 2014; Jordan et al., 2014) or higher self-relevant groups (Wood et al., 1996; Thibaut, 2017). In this situation, children may need more cognitive resources to distinguish between highly self-relevant recipients and lowly self-relevant recipients so that the effect of the degree of self-relevance may be decreased in children’s sharing.

## General Discussion

### More Self-Relevant Recipients, More Sharing: The Effect of the Degree of Self-Relevance on Preschool Children

Experiments 1 and 2 explored the impact of self-relevance on children’s sharing behaviors in first-party and third-party situations. The results of Experiment 1 and Experiment 2 both showed that children aged 3–6 could treat recipients differently based on self-relevance. In fact, the higher the self-relevance was, the more sharing that occurred among preschool children. These results were consistent with those of western studies. Previous studies found that preschool children tend to share more resources with friends than mere acquaintances (Costin and Jones, 1992; Rao and Stewart, 1999; Paulus and Moore, 2014) and share more with friends than with disliked peers, non-friends, or strangers (Birch and Billman, 1986; Fehr et al., 2008; Olson and Spelke, 2008). These studies showed that early sharing behavior is obviously influenced by the closeness and intimacy of relationships between oneself and recipients, and parochialism played an important role in early sharing behaviors (Bernhard et al., 2006). Therefore, the results of both experiments showed that preschool children could take the self-relevance of recipients into account. The self-relevance between children and recipients will have an impact on sharing behaviors and decisions.

### More Mature Self-Relevance Awareness Develops Gradually: The Effect of the Degree of Self-Relevance on Children’s Sharing Behavior Becomes Steadier With Age

The results in the non-costly, costly and envy conditions from Experiment 1 and those in the high self- and low self-bias situations from Experiment 2 showed that children aged 3–4 sometimes could not distinguish between moderately self-relevant and lowly self-relevant recipients and sometimes could not distinguish between highly self-relevant and moderately self-relevant recipients in terms of sharing behaviors. The self-concept and self-consciousness of children aged 3–4 are not mature enough, which leads to the unsteady effect of the degree of self-relevance in sharing behavior. However, children aged 5 to 6 were more likely to share according to the self-relevance of recipients in any situation observed in this study. As age increased, the self-concept and self-consciousness of children aged 5–6 tended to mature. Children could delicately process self-concept and differentiate among the three kinds of self-relevant recipients well. Therefore, the effect of the degree of self-relevance tends to be stable.

In recent years, most studies in psychology seem to be more inclined to support the concept of energy preservation rather than the concept of energy exhaustion (Muraven et al., 2006). For instance, Muraven asked all subjects to perform two different self-control tasks; however, before the second task, the experimental group was told that there was a third more important self-control tasks awaiting them; hence, the subjects in experimental group were observed to give up faster than those in the control group while performing the second self-control task, meaning that an individual might save certain self-control resources for a subsequent more important task (Muraven et al., 1998, 2006). Similar results were obtained in the study of Tyler and Burns (2008, 2009).

In Experiment 2, we examined the impact of self-control resources on deceptive behavior. Participants in the group of depletion of self-control resource had to complete a 15-min color discrimination Stroop task, then perform the operation test, and finally complete the red dot task. The control group participants only needed to complete a simple word recognition task (non-color noun), then perform the operation test, and, finally, finish the red dot task. The subjective assessment results showed that compared to the subjects in the control group, those in the group with depletion of the self-control resource believed that the task was more difficult; however, the differences in the evaluation of the level of effort by the two groups were not significant. These results might indicate that the subjects in the group of depletion of the self-control resource exhibited more deceptive behaviors or tendencies; however, in the previous task of self-control resource consumption (the Stroop task), the self-control resource was not depleted, and reserves of self-control resources were still available to cope with emergency incidents. Hence, our experiments also support the concept of energy preservation. Individuals fail to control themselves without completely depleting self-control resources, which is then followed by increased deceptive behaviors and tendencies.

### Strong Self-Interest Awareness: Preschool Children Are Willing to Share Resources in Unrelated Self-Interest Situations

The results from the non-costly situation in Experiment 1 and all results from the third-party situation in Experiment 2 showed a difference based on age. The non-costly condition of Experiment 1 provided two options, one to participants and zero to the recipients (1/0), or one to participants and one to the recipients (1/1). In the non-costly condition from experiment 1, participants would get 1 resource when they chose either of the two options and could not reduce or threaten their own self-interest. This condition is similar to all situations in experiment 2 since participants were not recipients of resources so that all options were unrelated to their self-interest. Research (Scharpf et al., 2017) from Uganda, a collective socialist country in Africa, adopted the same paradigm as our study and found that children aged 4–5 and children aged 6–7 also did not display an age difference in sharing. A cross-cultural study (Rochat et al., 2009) supposed that children growing up in a collectivist social culture are more likely to share to benefit others than those brought up in individualistic cultures, regardless of the age of the children. Chinese children live in a collectivist social culture and are educated based on a tradition of sharing and equity. Children aged 3–4 and aged 5–6 were both willing to share with others when sharing did not reduce their self-interest. Young children are sensitive to self-interest, and such self-interest influences children’s trust and trustworthiness (Reyes-Jaquez and Echols, 2015). Moreover, self-interest impacts children’s prosocial behaviors and decisions (Dietz, 2015; Zlatev and Miller, 2016). The results of this study provided more evidence of children’s awareness of self-interest. When under conditions related to self-interest in costly and envy situations, differences based on age appear in children’s sharing behaviors. These results may indicate that children aged 3–6 have a very strong awareness of self-interest when sharing resources.

## Ethics Statement

This study was approved by the ethics committee of Hunan Normal University.

## Author Contributions

WZ prepared the experimental procedures. WF and HD analyzed the data and wrote the manuscript. SX and YS performed the experimental procedures and organized participants to the experiments. WZ and MR contributed to the experimental materials. YZ reviewed the manuscript.

## Conflict of Interest Statement

The authors declare that the research was conducted in the absence of any commercial or financial relationships that could be construed as a potential conflict of interest.

## References

[B1] Aron A. Aron E. N. Smollan D. (1992). Inclusion of other in the self scale and the structure of interpersonal closeness. *J. Pers. Soc. Psychol.* 63 596–612. 10.1037//0022-3514.63.4.596

[B2] Bauer M. Cassar A. Chytilová J. Henrich J. (2014). War’s enduring effects on the development of egalitarian motivations and in-group biases. *Psychol. Sci.* 25 47–57. 10.1177/0956797613493444 24220626

[B3] Baumard N. Mascaro O. Chevallier C. (2012). Preschoolers are able to take merit into account when distributing goods. *Dev. Psychol.* 48 492–498. 10.1037/a0026598 22148948

[B4] Benenson J. F. Pascoe J. Radmore N. (2007). Children’s altruistic behavior in the dictator game. *Evol. Hum. Behav.* 28 168–175. 10.1016/j.jecp.2016.07.010 27552298

[B5] Bernhard H. Fischbacher U. Fehr E. (2006). Parochial altruism in humans. *Nature* 442:912. 10.1038/nature04981 16929297

[B6] Birch L. L. Billman J. (1986). Preschool children’s food sharing with friends and acquaintances. *Child Dev.* 57 387–395. 10.1111/j.1467-8624.1986.tb00038.x

[B7] Blake P. R. Mcauliffe K. (2011). “I had so much it didn’t seem fair”: Eight-year-olds reject two forms of inequity. *Cognition* 120 215–224. 21616483 10.1016/j.cognition.2011.04.006

[B8] Blake P. R. Mcauliffe K. Corbit J. Callaghan T. C. Barry O. Bowie A. (2015). Data from: the ontogeny of fairness in seven societies. *Nature* 528 258–261. 10.1038/nature15703 26580018

[B9] Blake P. R. Rand D. G. (2010). Currency value moderates equity preference among young children. *Evol. Hum. Behav.* 31 210–218.

[B10] Brown P. Keenan J. M. Potts G. R. (1986). The self-reference effect with imagery encoding. *J. Pers. Soc. Psychol.* 51 897–906. 11975152

[B11] Brownell C. A. Svetlova M. Nichols S. (2009). To share or not to share: when do toddlers respond to another’s needs? *Infancy* 14 117–130. 22639549 10.1080/15250000802569868 PMC3359011

[B12] Costin S. E. Jones D. C. (1992). Friendship as a facilitator of emotional responsiveness and prosocial interventions among young children. *Dev. Psychol.* 28 941–947.

[B13] Cowell J. M. Kang L. Malcolm-Smith S. Selcuk B. Zhou X. Decety J. (2017). The development of generosity and moral cognition across five cultures. *Dev. Sci.* 20 1–12. 10.1111/desc.12403 27146417

[B14] Crittenden A. N. Zes D. A. (2015). Food sharing among hadza hunter-gatherer children. *PLoS One* 10:e0131996. 10.1371/journal.pone.0131996 26151637 PMC4494808

[B15] Dietz T. (2015). Altruism, self-interest, and energy consumption. *Proc. Natl. Acad. Sci.* 112 1654–1655. 25587135 10.1073/pnas.1423686112 PMC4330781

[B16] Fan W. Chen J. Wang X. Y. Cai R. Tan Q. Chen Y. (2013). Electrophysiological correlation of the degree of self-reference effect. *PLoS One* 8:e80289. 10.1371/journal.pone.0080289 24312467 PMC3846566

[B17] Farb N. A. S. Segal Z. V. Mayberg H. Bean J. Mckeon D. Fatima Z. (2007). Attending to the present: mindfulness meditation reveals distinct neural modes of self-reference. *Soc. Cogn. Affect Neurosci.* 2 313–322. 10.1093/scan/nsm030 18985137 PMC2566754

[B18] Fehr E. Bernhard H. Rockenbach B. (2008). Egalitarianism in young children. *Nature* 454 1079–1083. 10.1038/nature07155 18756249

[B19] Fehr E. Schmidt K. M. (1999). *A Theory of Fairness, Competition, and Cooperation.* University of Munich: Munich.

[B20] Gasiorowska A. Chaplin L. N. Zaleskiewicz T. Wygrab S. Vohs K. D. (2016). Money cues increase agency and decrease prosociality among children early signs of market-mode behaviors. *Psychol. Sci.* 27 331–344. 10.1177/0956797615620378 26786823

[B21] Gerardi-Caulton G. (2000). Sensitivity to spatial conflict and the development of self-regulation in children 24–36 months of age. *Dev. Sci.* 3 397–404.

[B22] Gummerum M. Hanoch Y. Keller M. Parsons K. Hummel A. (2010). Preschoolers’ allocations in the dictator game: the role of moral emotions. *J. Econ. Psychol.* 31 25–34.

[B23] Gummerum M. Keller M. Takezawa M. Mata J. (2008). To give or not to give: children’s and adolescents’ sharing and moral negotiations in economic decision situations. *Child Develop.* 79 562–576. 10.1111/j.1467-8624.2008.01143.x. 18489413

[B24] Hao J. Yang Y. Wang Z. (2016). Face-to-Face sharing with strangers and altruistic punishment of acquaintances for strangers: young adolescents exhibit greater altruism than adults. *Front. Psychol.* 7:1512. 10.3389/fpsyg.2016.01512 27752246 PMC5045925

[B25] Hart S. L. Behrens K. Y. (2013). Affective and behavioral features of jealousy protest: associations with child temperament, maternal interaction style, and attachment. *Infancy* 18 369–399.

[B26] Jehn K. A. Shah P. P. (1997). Interpersonal relationships and task performance: an examination of mediation processes in friendship and acquaintance groups. *J. Pers. Soc. Psychol.* 72 775–790.

[B27] Jordan J. J. McAuliffe K. Warneken F. (2014). Development of in-group favoritism in children’s third-party punishment of selfishness. *Proc. Natl. Acad. Sci.* 111 12710–12715. 10.1073/pnas.1402280111 25136086 PMC4156744

[B28] Kanngiesser P. Warneken F. (2012). Young children consider merit when sharing resources with others. *PLoS One* 7:e43979. 10.1371/journal.pone.0043979 22952834 PMC3430625

[B29] Kenward B. Dahl M. (2011). Preschoolers distribute scarce resources according to the moral valence of recipients’ previous actions. *Dev. Psychol.* 47 1054–1064. 10.1037/a0023869 21604863

[B30] Kenward B. Hellmer K. Winter L. S. Eriksson M. (2015). Four-year-olds’ strategic allocation of resources: attempts to elicit reciprocation correlate negatively with spontaneous helping. *Cognition* 136 1–8. 25490123 10.1016/j.cognition.2014.11.035

[B31] Klein S. B. Loftus J. Schell T. (1994). Repeated testing: a technique for assessing the roles of elaborative and organizational processing in the representation of social knowledge. *J. Pers. Soc. Psychol.* 66 830–839.

[B32] Kogut T. Slovic P. Västfjäll D. (2015). The effect of recipient identifiability and neediness on children’s sharing behavior. *J. Behav. Decis. Mak.* 12 33–59.

[B33] Lobue V. Nishida T. Chiong C. Deloache J. S. Haidt J. (2011). When getting something good is bad: even three-year-olds react to inequality. *Soc. Dev.* 20 154–170.

[B34] Lu H. J. Chang L. (2016). Resource allocation to kin, friends, and strangers by 3- to 6-year-old children. *J. Exp. Child. Psychol.* 150 194–206. 10.1016/j.jecp.2016.05.018 27336694

[B35] Malti T. Gummerum M. Ongley S. Chaparro M. Nola M. Bae N. Y. (2016). Who is worthy of my generosity?” recipient characteristics and the development of children’s sharing. *Int. J Behav. Dev.* 40 31–40.

[B36] Markovits H. Benenson J. F. Kramer D. L. (2003). Children and adolescents’ internal models of food-sharing behavior include complex evaluations of contextual factors. *Child Dev.* 74 1697–1708. 14669890 10.1046/j.1467-8624.2003.00632.x

[B37] Masciuch S. Kienapple K. (1993). The emergence of jealousy in children 4 months to 7 years of age. *J. Soc. Pers. Relat.* 10 421–435.

[B38] Mei Haibo (2013). Self-Referential Effect of Three-Year-Old Children and Familiarity with Other People’s Reference Effects. Haidian: Peking University.

[B39] Mize K. D. Pineda M. Blau A. K. Marsh K. Jones N. A. (2014). Infant physiological and behavioral responses to a jealousy provoking condition. *Infancy* 19 338–348.

[B40] Moore C. (2009). Fairness in children’s resource allocation depends on the recipient. *Psychol. Sci.* 20 944–948. 10.1111/j.1467-9280.2009.02378.x 19515118

[B41] Mulvey K. L. Buchheister K. McGrath K. (2016). Evaluations of intergroup resource allocations: the role of theory of mind. *J. Exp. Child Psychol.* 142 203–211. 10.1016/j.jecp.2015.10.002 26525855

[B42] Muraven M. Shmueli D. Burkley E. (2006). Conserving self-control strength. *J. Pers. Soc. Psychol.* 91 524–537. 10.1037/0022-3514.91.3.524 16938035

[B43] Muraven M. Tice D. M. Baumeister R. F. (1998). Self-control as a limited resource: regulatory depletion patterns. *J. Pers. Soc. Psychol.* 74 774–789. 10.1037/0022-3514.74.3.774 9523419

[B44] Newcomb A. F. Bagwell C. L. (1995). Children’s friendship relations: a meta-analytic review. *Psychol. Bull.* 117 306–347.

[B45] Olson K. R. Spelke E. S. (2008). Foundations of cooperation in young children. *Cognition* 108 222–231. 10.1016/j.cognition.2007.12.003 18226808 PMC2481508

[B46] Pappert A. T. Williams A. Moore C. (2016). The influence of competition on resource allocation in preschool children. *Soc. Dev.* 26 367–381.

[B47] Paulus M. (2016a). Friendship trumps neediness: the impact of social relations and others’ wealth on preschool children’s sharing. *J. Exp. Child Psychol.* 146 106–120. 10.1016/j.jecp.2016.02.001 26930165

[B48] Paulus M. (2016b). It’s payback time: preschoolers selectively request resources from someone they had benefitted. *Dev. Psychol.* 52 1299–1306. 10.1037/dev0000150 27359157

[B49] Paulus M. Gillis S. Li J. Moore C. (2013). Preschool children involve a third party in a dyadic sharing situation based on fairness. *J. Exp. Child Psychol.* 116 78–85. 10.1016/j.jecp.2012.12.014 23597498

[B50] Paulus M. Licata M. Kristen S. Thoermer C. Woodward A. Sodian B. (2015). Social understanding and self-regulation predict pre-schoolers’ sharing with friends and disliked peers: a longitudinal study. *Int. J. Behav. Dev.* 39 53–64.

[B51] Paulus M. Moore C. (2014). The development of recipient-dependent sharing behavior and sharing expectations in preschool children. *Dev. Psychol.* 50 914–921. 10.1037/a0034169 23978297

[B52] Rao N. Stewart S. M. (1999). Cultural influences on sharer and recipient behavior: sharing in Chinese and Indian preschool children. *J. Cross Cult. Psychol.* 30 219–241.

[B53] Reyes-Jaquez B. Echols C. H. (2015). Playing by the rules: self-interest information influences children’s trust and trustworthiness in the absence of feedback. *Cognition* 134 140–154. 10.1016/j.cognition.2014.10.002 25460387

[B54] Rochat P. Dias M. D. G. Guo L. Broesch T. Passos-Ferreira C. Winning A. (2009). Fairness in distributive justice by 3- and 5-year-olds across seven cultures. *J. Cross Cult. Psychol.* 40 416–442.

[B55] Rogers T. B. Kuiper N. A. Kirker W. S. (1977). Self-reference and the encoding of personal information. *J. Pers. Soc. Psychol.* 35 677–688. 909043 10.1037//0022-3514.35.9.677

[B56] Scharpf F. Paulus M. Wörle M. (2016). The impact of social relationships on Ugandan children’s sharing decisions. *Eur. J. Dev Psychol.* 14 436–448.

[B57] Scharpf F. Paulus M. Wörle M. (2017). The impact of social relationships on Ugandan children’s sharing decisions. *Eur. J. Dev. Psychol.* 14 436–448.

[B58] Shaw A. Montinari N. Piovesan M. Olson K. R. Gino F. Norton M. I. (2014). Children develop a veil of fairness. *J. Exp Psychol. Gen.* 143:363. 10.1037/a0031247 23317084

[B59] Shaw A. Olson K. R. (2012). Children discard a resource to avoid inequity. *J. Exp. Psychol. Gen.* 141:382. 10.1037/a0025907 22004168

[B60] Sheskin M. Bloom P. Wynn K. (2014). Anti-equality: social comparison in young children. *Cognition* 130 152–156. 10.1016/j.cognition.2013.10.008 24291266 PMC3880565

[B61] Sheskin M. Nadal A. Croom A. Mayer T. Nissel J. Bloom P. (2016). Some equalities are more equal than others: quality equality emerges later than numerical equality. *Child Dev.* 87 1520–1528. 10.1111/cdev.12544 27142728

[B62] Smith C. E. Blake P. R. Harris P. L. (2013). I should but I won’t: why young children endorse norms of fair sharing but do not follow them. *PLoS One* 8:e59510. 10.1371/journal.pone.0059510 23527210 PMC3603928

[B63] Sparks E. Schinkel M. G. Moore C. (2017). Affiliation affects generosity in young children: the roles of minimal group membership and shared interests. *J. Exp. Child Psychol.* 159 242–262. 10.1016/j.jecp.2017.02.007 28327384

[B64] Steinbeis N. Over H. (2017). Enhancing behavioral control increases sharing in children. *J. Exp. Child Psychol.* 159 310–318. 10.1016/j.jecp.2017.02.001 28291515

[B65] Steinbeis N. Singer T. (2013). The effects of social comparison on social emotions and behavior during childhood: the ontogeny of envy and Schadenfreude predicts developmental changes in equity-related decisions. *J. Exp. Child Psychol.* 115 198–209. 10.1016/j.jecp.2012.11.009 23374608

[B66] Sui J. Zhu Y. (2005). Five-year-olds can show the self-reference advantage. *Int. J. Behav. Dev.* 29 382–387.

[B67] Svetlova M. Nichols S. R. Brownell C. A. (2010). Toddlers’ prosocial behavior: from instrumental to empathic to altruistic helping. *Child Dev.* 81:1814. 10.1111/j.1467-8624.2010.01512.x 21077866 PMC3088085

[B68] Symons C. S. Johnson B. T. (1997). The self-reference effect in memory: a meta-analysis. *Psychol. Bull.* 121 371–394. 9136641 10.1037/0033-2909.121.3.371

[B69] Tan Q. Zhan Y. Gao S. Fan W. Chen J. Zhong Y. (2015). Closer the relatives are, more intimate and similar we are: kinship effects on self-other overlap. *Pers. Individ. Diff.* 73 7–11.

[B70] Thibaut J. W. (2017). *The Social Psychology of Groups.* Abingdon: Routledge.

[B71] Thompson C. Barresi J. Moore C. (1997). The development of future-oriented prudence and altruism in preschoolers. *Cogn. Dev.* 12 199–212.

[B72] Tyler J. M. Burns K. C. (2008). After depletion: the replenishment of the self’s regulatory resources. *Self Identity* 7 305–321. 10.1002/ijop.12235 26564863

[B73] Tyler J. M. Burns K. C. (2009). Triggering conservation of the self’s regulatory resources. *Basic Appl. Soc. Psychol.* 31 255–266. 10.1080/01973530903058490

[B74] Ugurel-Semin R. (1952). Moral behavior and moral judgment of children. *J Abnorm. Psychol.* 47 (Suppl.2) 463–474. 14937990 10.1037/h0056970

[B75] Wilkening F. (2009). Children’s and adolescents’ intuitive judgements about distributive justice: integrating need, effort, and luck. *Eur. J. Dev. Psychol.* 6 481–498.

[B76] Williams A. O’Driscoll K. Moore C. (2014). The influence of empathic concern on prosocial behavior in children. *Front. Psychol.* 5:425. 10.3389/fpsyg.2014.00425 24860537 PMC4026684

[B77] Wood W. Pool G. J. Leck K. Purvis D. (1996). Self-definition, defensive processing, and influence: the normative impact of majority and minority groups. *J. Pers. Soc. Psychol.* 71 1181–1193. 8979385 10.1037//0022-3514.71.6.1181

[B78] Xiong M. Shi J. Wu Z. Zhang Z. (2016). Five-year-old preschoolers’ sharing is influenced by anticipated reciprocation. *Front. Psychol.* 7:460. 10.3389/fpsyg.2016.00460 27064475 PMC4814498

[B79] Zhong Y. Yang Z. Fan W. (2015). The influence of self-others’ overlap on helping people’s behavior: the role of perspective selection. *Acta Psychol. Sin.* 47 1050–1057.

[B80] Zhou H. Guo J. Ma X. Zhang M. Liu L. Feng L. (2017). Self-reference emerges earlier than emotion during an implicit self-referential emotion processing task: event-related potential evidence. *Front. Hum. Neurosci.* 11:451. 10.3389/fnhum.2017.00451 28943845 PMC5596083

[B81] Zhou A. Liu P. Shi Z. Zhang P. Wu H. Li Q. (2010). Self-referential effect of four-year-old children. *Psychol. Dev. Educ.* 26 239–244.

[B82] Zlatev J. J. Miller D. T. (2016). Selfishly benevolent or benevolently selfish: when self-interest undermines versus promotes prosocial behavior. *Organ. Behav. Hum Decis. Process.* 137 112–122. 9873976

